# Manipulation of Saliva-Derived Microcosm Biofilms To Resemble Dysbiotic Subgingival Microbiota

**DOI:** 10.1128/AEM.02371-20

**Published:** 2021-01-15

**Authors:** Yaling Jiang, Bernd W. Brandt, Mark J. Buijs, Lei Cheng, Rob A. M. Exterkate, Wim Crielaard, Dong Mei Deng

**Affiliations:** a State Key Laboratory of Oral Diseases & National Clinical Research Center for Oral Diseases & Department of Cariology and Endodontics, West China Hospital of Stomatology, Sichuan University, Chengdu, China; b Department of Preventive Dentistry, Academic Center for Dentistry Amsterdam (ACTA), University of Amsterdam and Vrije Universiteit Amsterdam, Amsterdam, The Netherlands; University of Tokyo

**Keywords:** *Porphyromonas gingivalis*, oral microbiome, subgingival, biofilm model, microcosm, 16S rRNA genes, periodontitis, butyric acid, dipeptidyl peptidase IV

## Abstract

Periodontitis is a highly prevalent oral inflammatory disease triggered by dysbiotic subgingival microbiota. For the development of microbiome modulators that can reverse the dysbiotic state and reestablish a health-associated microbiota, a high-throughput *in vitro* multispecies biofilm model is needed. Our aim is to establish a model that resembles a dysbiotic subgingival microbial biofilm by incorporating the major periodontal pathogen Porphyromonas gingivalis into microcosm biofilms cultured from pooled saliva of healthy volunteers. The biofilms were grown for 3, 7, and 10 days and analyzed for their microbial composition by 16S rRNA gene amplicon sequencing as well as measurement of dipeptidyl peptidase IV (DPP4) activity and butyric acid production. The addition of P. gingivalis increased its abundance in saliva-derived microcosm biofilms from 2.7% on day 3 to >50% on day 10, which significantly reduced the Shannon diversity but did not affect the total number of operational taxonomic units (OTUs). The P. gingivalis-enriched biofilms displayed altered microbial composition as revealed by principal-component analysis and reduced interactions among microbial species. Moreover, these biofilms exhibited enhanced DPP4 activity and butyric acid production. In conclusion, by adding P. gingivalis to saliva-derived microcosm biofilms, we established an *in vitro* pathogen-enriched dysbiotic microbiota which resembles periodontitis-associated subgingival microbiota in terms of increased P. gingivalis abundance and higher DPP4 activity and butyric acid production. This model may allow for investigating factors that accelerate or hinder a microbial shift from symbiosis to dysbiosis and for developing microbiome modulation strategies.

**IMPORTANCE** In line with the new paradigm of the etiology of periodontitis, an inflammatory disorder initiated by dysbiotic subgingival microbiota, novel therapeutic strategies have been proposed targeting reversing dysbiosis and restoring host-compatible microbiota rather than eliminating the biofilms unselectively. Thus, appropriate laboratory models are required to evaluate the efficacy of potential microbiome modulators. In the present study, we used the easily obtainable saliva as an inoculum, spiked the microcosm biofilms with the periodontal pathogen Porphyromonas gingivalis, and obtained a P. gingivalis-enriched microbiota, which resembles the *in vivo* pathogen-enriched subgingival microbiota in severe periodontitis. This biofilm model circumvents the difficulties encountered when using subgingival plaque as the inoculum and achieves microbiota in a dysbiotic state in a controlled and reproducible manner, which is required for high-throughput and large-scale evaluation of strategies that can potentially modulate microbial ecology.

## Abstract (precis)

In line with the new paradigm of the etiology of periodontitis, an inflammatory disorder initiated by dysbiotic subgingival microbiota, novel therapeutic strategies have been proposed targeting reversing dysbiosis and restoring host-compatible microbiota rather than eliminating the biofilms unselectively. Thus, appropriate laboratory models are required to evaluate the efficacy of potential microbiome modulators.

## INTRODUCTION

Periodontitis is a highly prevalent inflammatory oral disease affecting over 740 million people worldwide (1). It is characterized by progressive destruction of the tooth-supporting tissues, hence representing a major cause of tooth loss in adults (2). Furthermore, periodontitis has been adversely implicated in a number of systemic diseases, such as cardiovascular diseases and diabetes (3). Traditionally, the development of periodontitis is linked to a few pathogens, notably members of the “red complex” (Porphyromonas gingivalis, Tannerella forsythia, and Treponema denticola) (4). Recent studies based on next-generation sequencing technologies have uncovered that periodontitis is not related to a single or several pathogens. In contrast, periodontitis is a complex microbial community-associated disease, initiated by a deleterious transition from a symbiotic to a pathogen-enriched dysbiotic state (5, 6).

In line with the paradigm shift of the etiology of periodontitis, novel therapeutic strategies have been proposed, aiming for reversing dysbiosis and restoring and maintaining host-microbiome balance rather than eliminating the biofilms unselectively (7). A good example has been seen in the management of dental caries, another infectious disease caused by the dysbiosis of oral microbiota. The application of arginine-containing toothpaste has been shown to be able to shift the oral microbiota of caries-active patients to healthier communities, which had a bacterial composition similar to that of caries-free individuals (8, 9). Recently, Adams et al. (10) reported that the use of a toothpaste containing enzymes (amyloglucosidase, glucose oxidase, and lactoperoxidase) and proteins (lysozyme, lactoferrin, and IgG) led to an increase in microbes associated with gingival health but a decrease in those associated with periodontal disease. However, only healthy subjects were examined in the latter study, and the ability of this novel microbiome modulator to reverse a microbiome in the dysbiotic state needs further evaluation.

The development and evaluation of novel microbiome modulators require suitable *in vitro* multispecies biofilm models. These cultured biofilms should resemble the microbiota shift from healthy symbiotic to a pathogen-enriched dysbiotic state. The efficacy of a modulator in maintaining the healthy microbial ecology or reversing the dysbiotic state could then be evaluated in these models before starting clinical trials. Various *in vitro* multispecies biofilm models have been developed in recent years. Many of them were designed to simulate the complex subgingival microbiota (11–14). Using subgingival plaque specimens from periodontitis patients as inocula, these models were able to produce diverse and pathogen-enriched microcosm biofilms which mimicked the complexity of *in vivo* periodontitis-associated subgingival microbiota. However, there are limitations when using subgingival plaque as an inoculum, including (i) it requires patient selection and specialized training for proper sampling; and (ii) the amount of plaque obtained is often limited, which restricts the number of biofilm samples that can be cultured in one experiment. In light of this, Cieplik et al. (15) have examined the possibility of using the easily obtainable saliva from periodontitis patients as an inoculum. Unfortunately, the known periodontal pathogens were barely found in these saliva-derived microcosm biofilms. However, Naginyte et al. (13), using pooled saliva, plaque, and tongue samples from dentally healthy subjects as the inoculum, claimed that periodontal pathogens could be enriched in *in vitro* biofilms, probably due to the use of serum-containing growth media, which simulated nutritional aspects of the subgingival environment (11).

Although saliva alone might not be a suitable inoculum for culturing pathogen-enriched subgingival biofilms according to the aforementioned study (15), the noninvasive accessibility and easy availability of saliva still make it an attractive source. Therefore, this study used pooled saliva from healthy subjects as the inoculum. By adding the major periodontal pathogen P. gingivalis into preformed microcosm biofilms and using a serum-rich medium, we aimed to develop an *in vitro* pathogen-enriched dysbiotic subgingival biofilm. This is a proof-of-principle study; hence, only one representative periodontal pathogen was added to examine the feasibility of the model.

## RESULTS

### Total viable cell counts.

**FIG 1:**
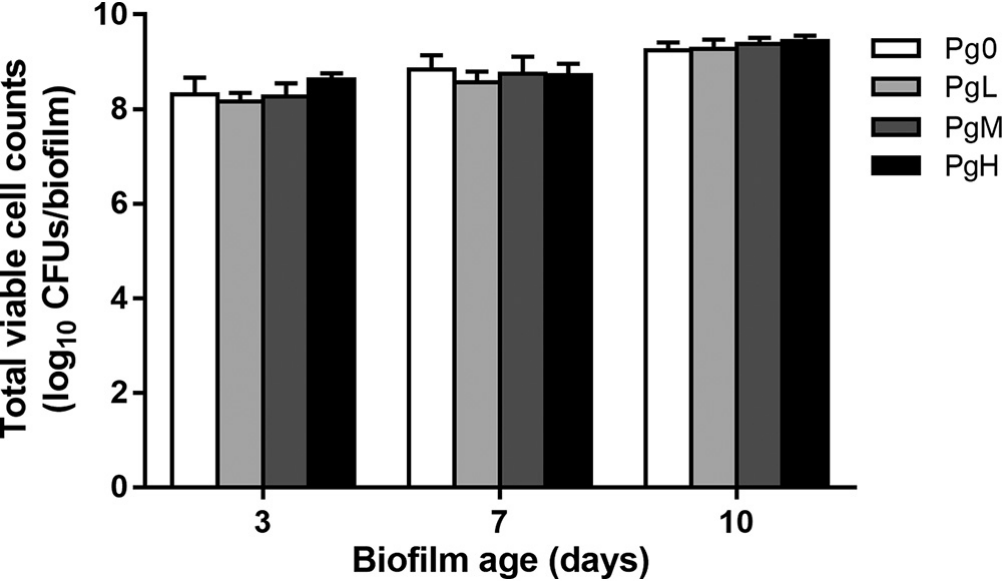
Total viable cell counts of the biofilms in different groups. Pg0, biofilms without the addition of P. gingivalis; PgL, PgM, and PgH, biofilms with the addition of low, medium, and large amounts of P. gingivalis, respectively.

Figure 1 shows the total viable cell counts of the microcosm biofilms. Generally, the total viable cell counts increased significantly with the increasing of biofilms’ age, irrespective of the addition of P. gingivalis. Although the initial amounts of P. gingivalis added to PgL, PgM, and PgH groups were 10-fold different, the total viable cell counts of the biofilms at each sample collection point were similar among different groups.

### The amount of P. gingivalis in microcosm biofilms.

**FIG 2:**
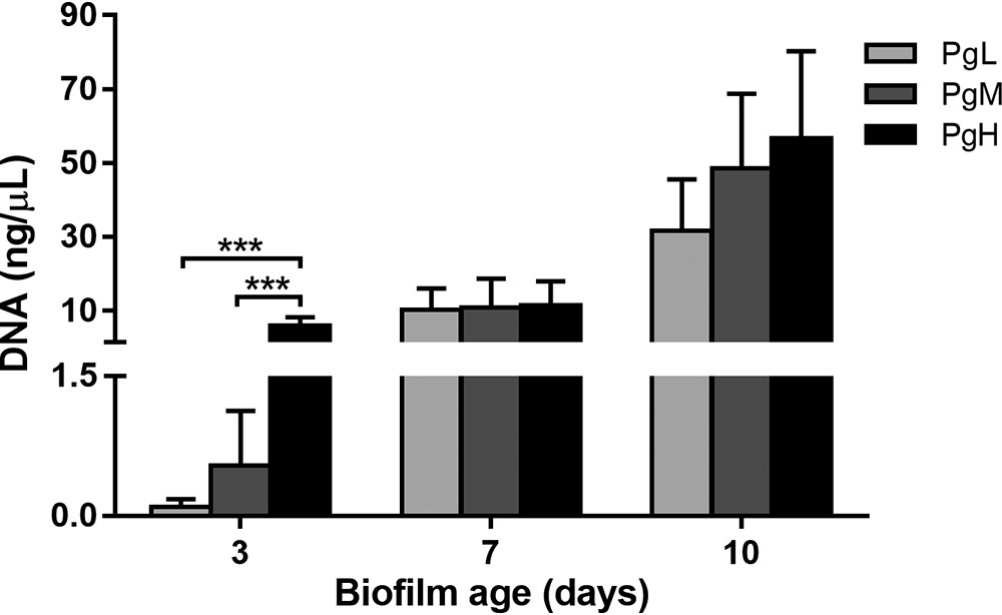
Amount of P. gingivalis in the biofilms as determined by qPCR. Data are presented as the calculated DNA concentration (nanograms per microliter) of P. gingivalis. PgL, PgM, and PgH, biofilms with the addition of low, medium, and large amounts of P. gingivalis, respectively. *** (*P < *0.0001), statistically significant differences.

A species-specific quantitative PCR (qPCR) was performed to quantify the amount of P. gingivalis in the biofilms (Fig. 2). P. gingivalis was detected in all biofilms to which P. gingivalis was added, and the amount generally increased with the increasing of biofilm age. On day 3, the amount of P. gingivalis detected in biofilms correlated with its initial inoculation amount. It was the highest in PgH biofilms (5.90 ± 2.25 ng/μl) and was the lowest in PgL biofilms (0.11 ± 0.07 ng/μl). However, this difference among the PgL, PgM, and PgH groups disappeared on days 7 and 10. In the Pg0 group, no P. gingivalis could be detected in the biofilms throughout the whole experimental period, although a trace amount of P. gingivalis was detected in the saliva inoculum (0.02 ± 0.01 ng/μl).

### Relative abundance of major bacterial genera established in the biofilms and α-diversity analyses of the biofilms.

**FIG 3:**
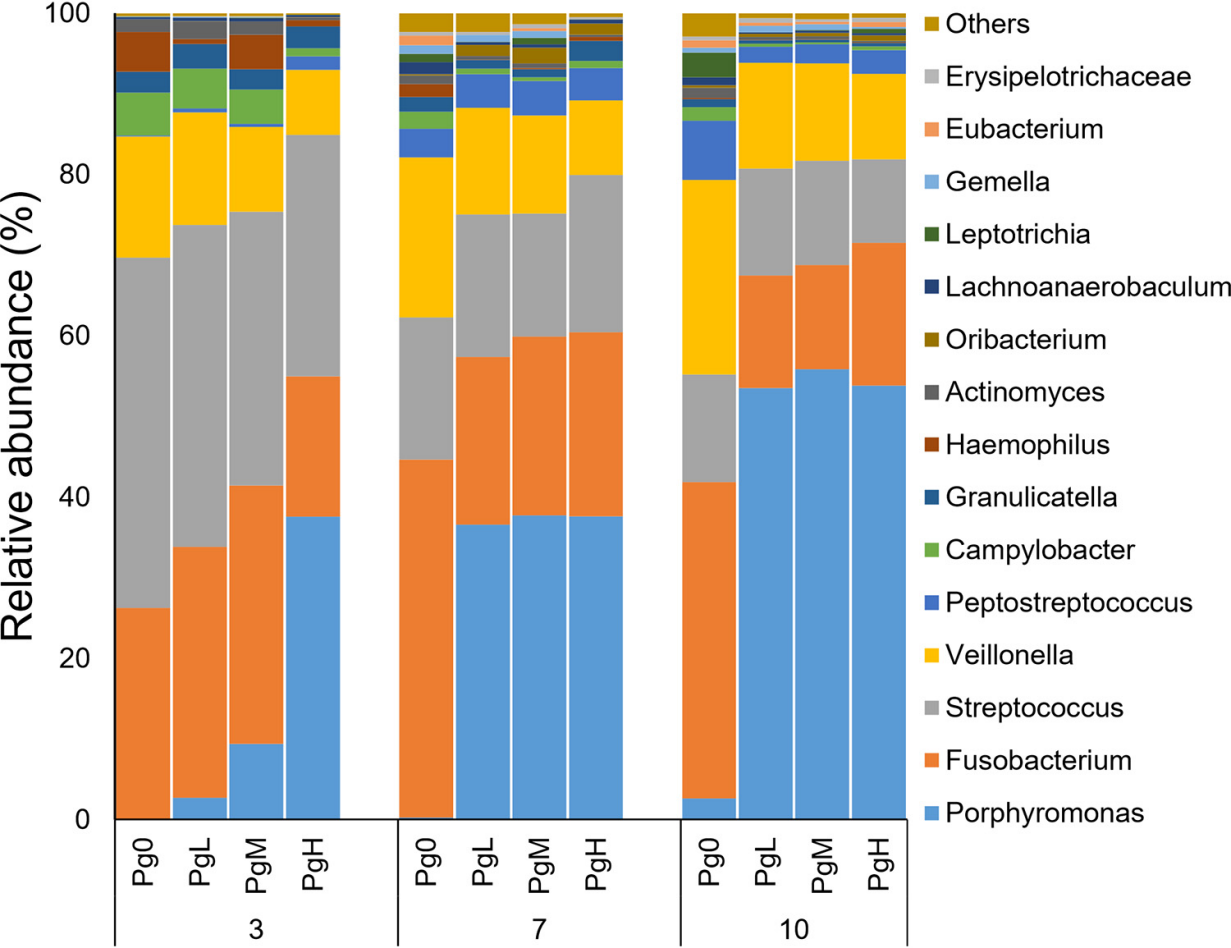
Relative abundance (average of replicates) of the top 15 most abundant bacteria genera or higher taxa (remaining genera are grouped as “others”) in the biofilms. Biofilms of the same age among different groups are plotted as adjacent bars. Pg0, biofilms without the addition of P. gingivalis; PgL, PgM, and PgH, biofilms with the addition of low, medium, and large amounts of P. gingivalis, respectively.

Figure 3 shows the average relative abundance of the top 15 most abundant bacterial genera or higher taxa in different groups. All biofilms were dominated by *Fusobacterium*, *Streptococcus*, and *Veillonella*. Compared to the Pg0 group, *Porphyromonas* became a predominant genus in the PgL, PgM, and PgH groups; its abundance increased from 2.7% in the PgL group on day 3 to >50% in PgL, PgM, and PgH groups on day 10. The average relative abundances of *Porphyromonas* on day 10 in the PgL, PgM, and PgH groups were, respectively, 53.5% ± 5.7% (coefficient of variation [CV], 11%), 55.9% ± 2.67% (CV, 5%), and 53.8% ± 6.5% (CV, 12%). The relatively low CV values indicate the enrichment of P. gingivalis is reproducible among repeated experiments. Concomitant with the increase in the relative abundance of *Porphyromonas*, the relative abundance of other major genera, including *Fusobacterium* and *Streptococcus*, decreased in time in these three groups. The genus *Porphyromonas* was also detected in Pg0 biofilms on day 3, with its abundance reaching 2.6% on day 10.

**FIG 4:**
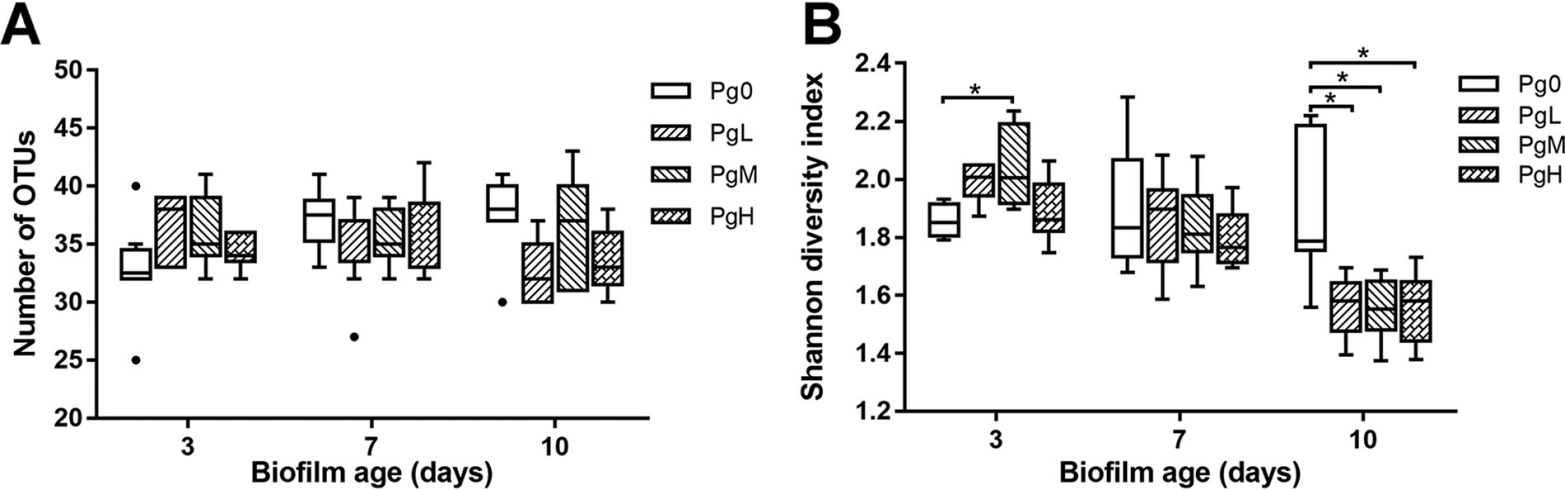
The α-diversity analyses of the biofilms in different groups using the observed number of OTUs (A) and the Shannon diversity index (B). The box plots are plotted using Tukey’s method. Pg0, biofilms without the addition of P. gingivalis; PgL, PgM, and PgH, biofilms with the addition of low, medium, and large amounts of P. gingivalis, respectively. * (*P < *0.01), statistically significant differences.

The α-diversity of the biofilms, indicated by the number of operational taxonomic units (OTUs) and Shannon diversity index, was further analyzed (Fig. 4). The number of OTUs in the saliva inoculum was 133 ± 5, while the average number of OTUs in biofilms of all ages was 35 ± 4. Comparing the number of OTUs in the PgL, PgM, and PgH groups to that of the Pg0 group at each time point, no statistically significant differences were found. However, the changes in the number of OTUs in time were different comparing PgL, PgM, and PgH groups to the Pg0 group. The number of OTUs significantly increased from day 3 to day 10 in the Pg0 group but remained unchanged in the PgL, PgM, and PgH groups. The Shannon diversity showed a result similar to that of the number of OTUs; the biofilms also exhibited a reduced diversity (1.82 ± 0.21) compared to the saliva inoculum (3.08 ± 0.03). However, the Shannon diversity of the PgL, PgM, and PgH groups was significantly lower than that of Pg0 group on day 10, whereas there was no significant difference in the number of OTUs. We also analyzed the Shannon diversity index after excluding the OTU (OTU1) representing the added P. gingivalis. In this case, the Shannon diversity of PgL, PgM, and PgH groups was similar to that of the Pg0 group and remained unchanged throughout the experiment (data not shown).

### Microbial composition of the biofilms.

**FIG 5:**
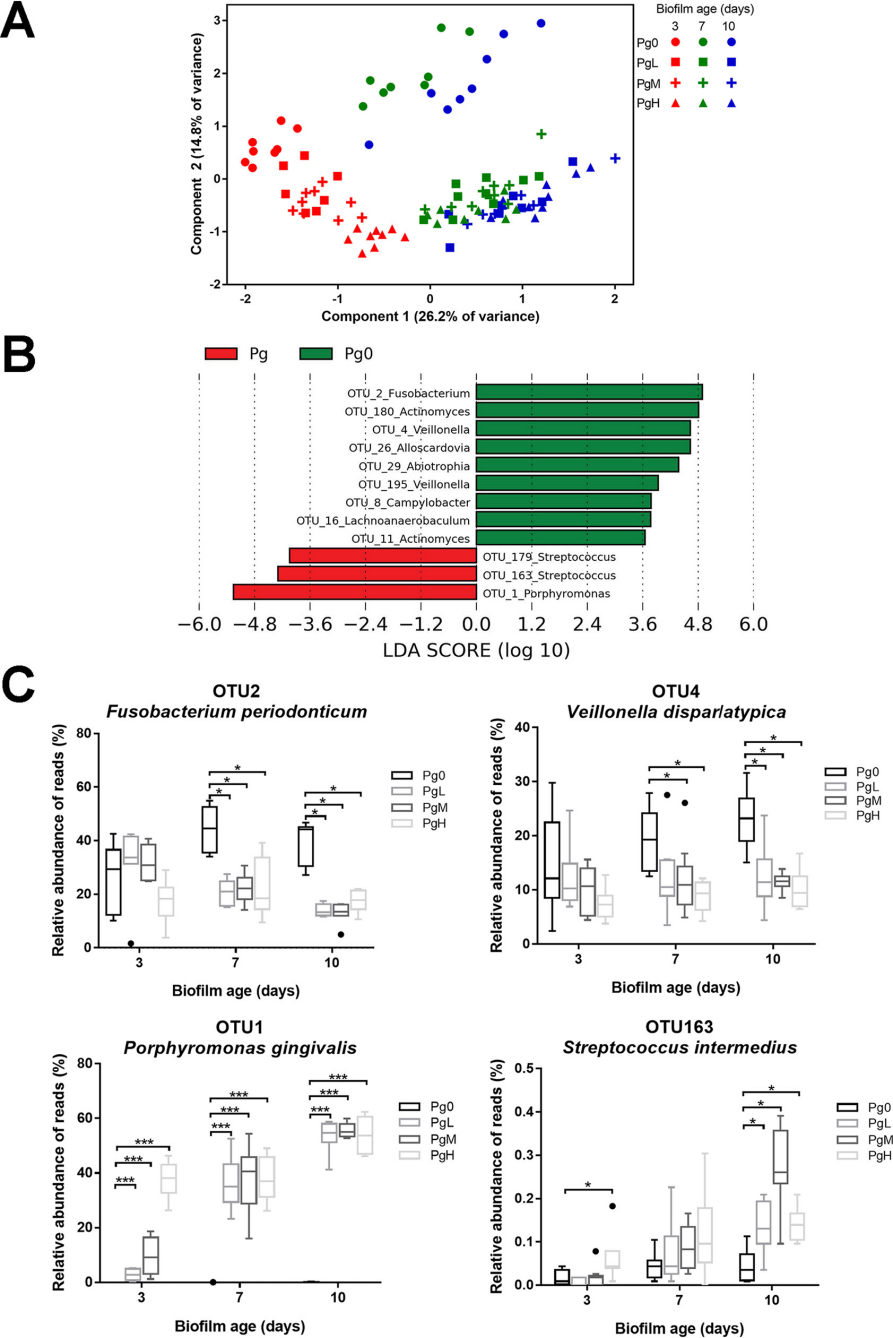
(A) Principal-component analysis (PCA) plot of Pg0, PgL, PgM, and PgH biofilms on day 3 (symbols in red), day 7 (symbols in green), and day 10 (symbols in blue). (B) OTUs that were differentially abundant between Pg0 biofilms (green bars) and Pg biofilms (red bars) in LEfSe. PgL, PgM, and PgH biofilms were combined as Pg biofilms, and biofilm age was set as subclass in the analysis. The taxonomy name of each OTU was given at genus or higher level. (C) Boxplots of the relative abundance of selected OTUs that were differentially abundant in biofilms with or without the addition of P. gingivalis (*, *P < *0.01, and ***, *P < *0.0001; Mann-Whitney test). Species names of the OTUs are obtained by blasting the representative sequences to the HOMD database.

The microbial profiles were ordinated in a principal-component analysis (PCA) plot (Fig. 5A) to visualize the microbial composition changes in biofilms. The PCA plot showed that all Pg biofilms (PgL, PgM, and PgH) clustered separately from Pg0 biofilms from day 3 onward. The separation was more evident on day 7 and day 10 as a result of the shift of Pg0 biofilms toward the upper right direction and Pg biofilms toward the right. The microbial compositions of biofilms in different groups were compared at each time point separately by one-way permutational multivariate analysis of variance (PERMANOVA) analysis using the Bray-Curtis similarity index, which confirmed that the addition of P. gingivalis led to significant changes in the microbial composition, irrespective of the biofilms’ age (Table 1).

**TABLE 1:** Bonferroni-corrected *P* and *F* values from the pairwise comparison in one-way PERMANOVA analysis^*a*^*^,^*^*b*^

Biofilm age (days)	*P* (*F*) for:					
	Pg0 vs PgL	Pg0 vs PgM	Pg0 vs PgH	PgL vs PgM	PgL vs PgH	PgM vs PgH
3	0.002^*c*^ (2.40)	0.003^*c*^ (3.39)	0.001^*c*^ (6.47)	1.000 (0.77)	0.001^*c*^ (6.47)	0.001^*c*^ (4.80)
7	0.001^*c*^ (7.04)	0.001^*c*^ (6.62)	0.002^*c*^ (9.32)	1.000 (0.69)	0.052 (2.18)	0.117 (2.01)
10	0.002^*c*^ (6.75)	0.002^*c*^ (5.71)	0.001^*c*^ (8.36)	1.000 (0.64)	0.212 (2.15)	0.651 (1.70)

a Biofilms in different groups were compared at each time point separately.

b Pg0, biofilms without the addition of P. gingivalis; PgL, PgM, and PgH, biofilms with the addition of low, medium, and large amounts of P. gingivalis, respectively.

c Statistically significant difference (*P < *0.01).

In addition, to assess the reproducibility of the biofilms in each group, paired comparisons of Bray-Curtis similarity indices between biofilm samples from the three independent experiments were analyzed. In general, a high Bray-Curtis similarity index between biofilms from independent experiments in each test condition was observed (see Table S1 in the supplemental material), ranging from 0.75 ± 0.07 to 0.84 ± 0.03, which indicates that the microbial compositions of the biofilms are highly reproducible among repeated experiments.

Linear discriminant analysis effect size (LEfSe) analysis was used to identify OTUs that were differentially abundant between Pg0 and Pg biofilms (Fig. 5B). *Fusobacterium* (OTU2), *Actinomyces* (OTU180 and OTU11), and *Veillonella* (OTU4 and OTU195) were mainly present in Pg0 biofilms, whereas *Porphyromonas* (OTU1) and *Streptococcus* (OTU163 and OTU179) were mainly present in the Pg biofilms. Among these OTUs, the relative abundance of the top two OTUs with the largest mean abundance over time was also plotted (Fig. 5C), and the species names of the OTUs were determined by blasting the representative sequences to the HOMD database. P. gingivalis (OTU1) was enriched in all Pg biofilms and increased in time. The representative sequence was indeed identical to that of strain ATCC 33277, which was added to the biofilms. Together with OTU1, the abundance of Streptococcus intermedius (OTU163) also increased in time in Pg biofilms. In contrast, the abundance of Fusobacterium periodonticum (OTU2) and Veillonella dispar/Veillonella atypica (OTU4) clearly decreased in Pg biofilms. *Porphyromonas* detected in Pg0 biofilms, as mentioned above, was also explored, which turned out to consist only of OTU21, identified as Porphyromonas pasteri, rather than P. gingivalis.

### Network analysis of biofilms with or without the addition of P. gingivalis.

**FIG 6:**
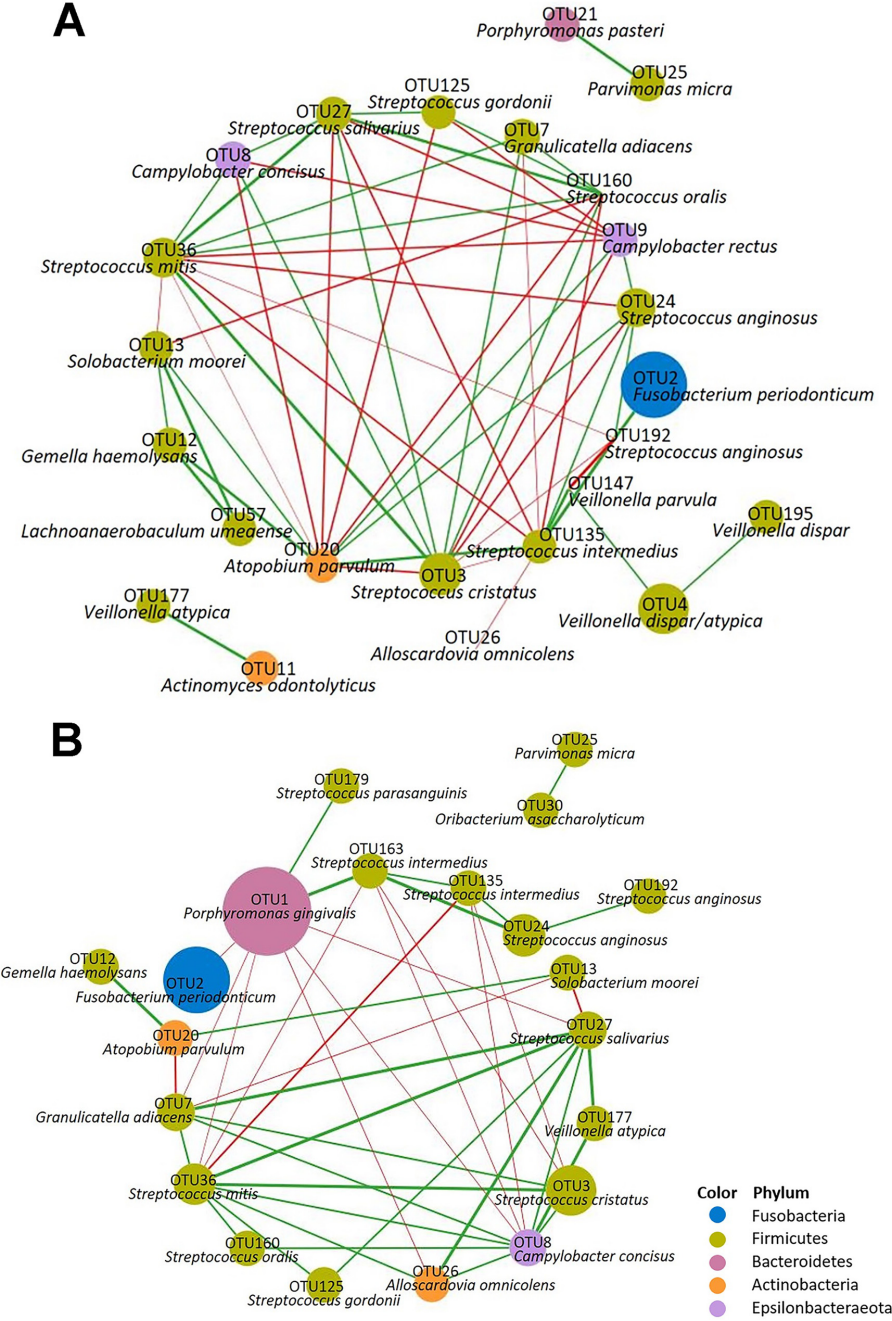
Microbial correlation networks in biofilms without the addition of P. gingivalis (Pg0 biofilms of days 3, 7, and 10) (A) and with the addition of P. gingivalis (PgL, PgM, and PgH biofilms of days 3, 7, and 10) (B). Only significant correlations (*P < *0.01) with Spearman |R| > 0.65 are presented. The edges represent copresence (green line) or mutual exclusion (red line) patterns among connected bacteria, and the line thickness reflects the absolute correlation value. The size of the nodes is proportional to the abundance of each OTU, and the color of the nodes indicates their classification at the phylum level. Species names of the OTUs are obtained by blasting the representative sequences to the HOMD database.

Spearman correlation networks were constructed to assess and visualize the mutual correlations among the OTUs in the Pg0 and Pg biofilms separately. Copresence and mutually excluded OTUs were indicated by nodes and connected by lines (edges) in the networks (Fig. 6). The network of Pg0 biofilms consisted of 24 nodes and 56 edges (copresence, 33; mutual exclusion, 23), whereas Pg biofilms showed a network with fewer connections, consisting of 21 nodes and 42 edges (copresence, 27; mutual exclusion, 15). The average number of neighbors in the Pg0 network (4.67) was slightly higher than that of Pg biofilms (4.00), indicating reduced connectivity resulting from the addition of P. gingivalis. In Pg0 biofilms, Streptococcus mitis (OTU36), Atopobium parvulum (OTU20), and Streptococcus cristatus (OTU3) had the most correlations with others, and in Pg biofilms, S. mitis (OTU36), Campylobacter concisus (OTU8), and P. gingivalis (OTU1) had the most connections. The added P. gingivalis (OTU1) mostly showed mutual exclusion correlations with others; it only positively correlated with Streptococcus parasanguinis (OTU179) and S. intermedius (OTU163), which supported the findings from LEfSe analysis (Fig. 5B).

### DPP4 activity and butyric acid production in the biofilms.

**FIG 7:**
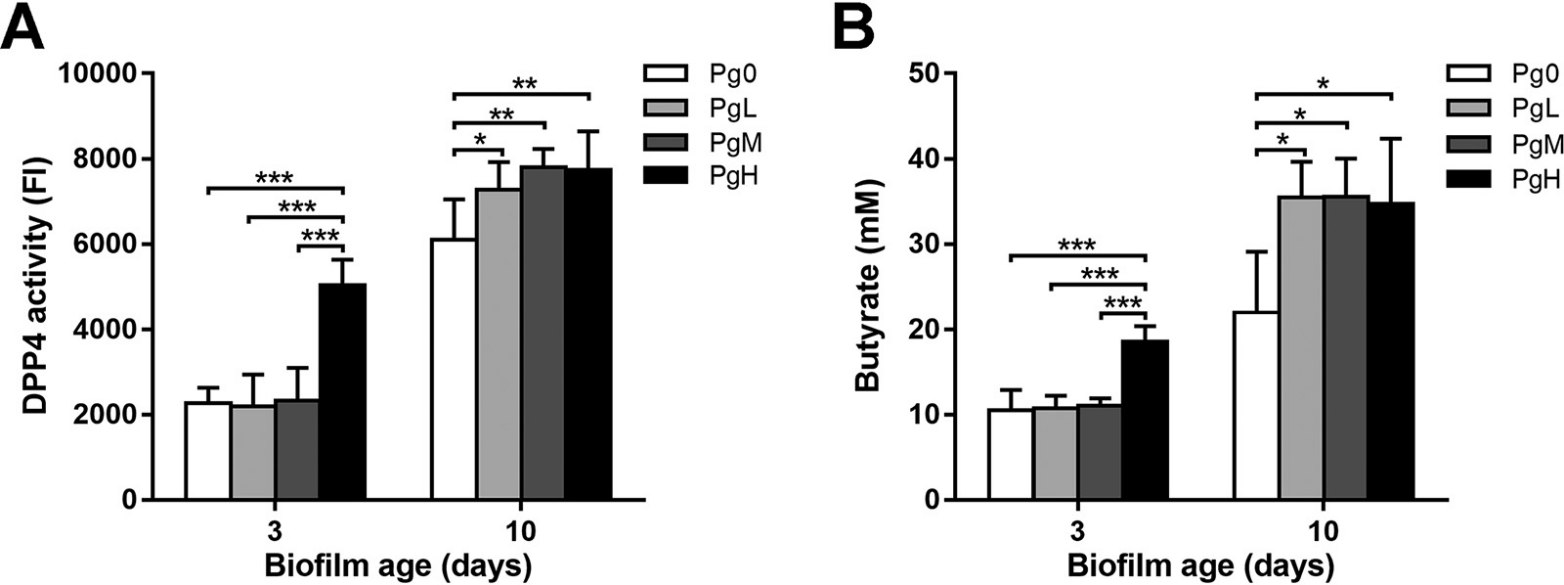
(A) DPP4 activity of biofilms. (B) Butyric acid production in biofilms, represented by the butyrate concentration in the biofilm spent medium. Pg0, biofilms without the addition of P. gingivalis; PgL, PgM, and PgH, biofilms with the addition of low, medium, and large amounts of P. gingivalis, respectively. * (*P < *0.01), ** (*P < *0.001), and *** (*P < *0.0001), statistically significant differences.

Both dipeptidyl peptidase IV (DPP4) activity and butyric acid production have been used as biomarkers to indicate the severity of periodontitis clinically (16, 17). Figure 7A shows that on day 3, PgH biofilms exhibited the highest DPP4 activity, whereas the other groups (PgL, PgM, and Pg0 biofilms) showed similar DPP4 activities. On day 10, DPP4 activities increased significantly in all biofilms compared to day 3. However, PgL, PgM, and PgH biofilms had significantly higher activities than Pg0 biofilms. Likewise, butyric acid production in the biofilms was also affected by the addition of P. gingivalis (Fig. 7B). The concentration of butyrate was significantly higher in PgH biofilms than in other biofilms on day 3, and it increased in all biofilms on day 10, with significantly higher butyrate concentrations in PgL, PgM, and PgH biofilms than in Pg0 biofilms.

## DISCUSSION

It has become widely accepted that periodontitis is a disease associated not with just a few pathogens but with a complex microbial community. Periodontitis is the result of an ecological shift of this community from symbiosis to a pathogen-enriched dysbiotic state (18). Along with this paradigm change, the need for the development of novel intervention strategies that could modulate the ecological state of the microbial community has become evident. Hence, a high-throughput *in vitro* biofilm model that can resemble the shift in microbial composition and/or represent one of the ecological states is desirable. By using the easily obtainable human saliva as an inoculum and supplementing the model with additional P. gingivalis, this study presented such a high-throughput and reproducible biofilm model that resembles a pathogen-enriched subgingival microbiota. In this model, P. gingivalis demonstrated the ability to integrate into a 24-h preformed microcosm biofilm. Its abundance in the microcosm biofilms increased from 2.7% to more than 50% in 10 days. It is worth noting that despite the high abundance of P. gingivalis in this model, the species richness (the number of OTUs) of the community did not decrease much, indicating the multispecies nature of the community was maintained.

The P. gingivalis-enriched microcosm biofilms established in this study resemble the dysbiotic subgingival microbiota in severe periodontitis. First of all, in clinical studies, increased relative abundances of P. gingivalis in subgingival microbiota have been positively correlated with the severity of periodontitis (5, 19). In the case of severe periodontitis, the relative abundance of P. gingivalis in subgingival microbiota could reach as high as 50% (20, 21), which is the same as demonstrated in this study (P. gingivalis-enriched biofilms on day 10). Second, in terms of the α-diversity of the *in vivo* subgingival microbiota, inconsistent results, however, have been reported in previous studies. Some studies reported it was higher than that of the healthy microbiota, but others reported no difference or a lower value (5, 22, 23). Although it is not feasible to directly compare our findings to the clinical findings observed under various disease conditions, it seems that the Shannon diversity, one of the α-diversity indices, decreased in the subgingival microbiota in severe periodontitis (20, 21), and the significantly lower Shannon diversity observed in the current P. gingivalis-enriched microcosm biofilms coincides with this trend. The reduced Shannon diversity with unchanged species richness in our study indicated a lower evenness in the microbial community, which is likely caused by the high proportion of P. gingivalis. Third, the network analysis showed the addition of P. gingivalis reduced the number of correlations between microbial species, which is in line with the findings in a previous clinical study (24). In that study, the total number of correlated bacterial species in the subgingival plaque from patients with progressive periodontitis was significantly less than that in the healthy samples. Overall, our results indicate that the P. gingivalis-enriched microcosm biofilms resemble the dysbiotic subgingival microbiota in severe periodontitis in terms of high P. gingivalis abundance, lower Shannon diversity, and reduced connectivity of the microbiota.

In addition to microbial composition, we also examined the physiological function of the biofilms with two clinical periodontal biomarkers, DPP4 and butyric acid. Our intention is to monitor the activities of these two biomarkers in the multispecies biofilms when P. gingivalis has incorporated into the biofilm and to examine if the function of the P. gingivalis-enriched microcosm biofilms, in terms of DPP4 activity and butyric acid production, can reflect that of the dysbiotic subgingival microbiota. DPP4 is a serine protease which cleaves X-Pro or X-Ala dipeptides from the N-terminal end of polypeptide chains, leading to the breakdown of periodontal tissues. It is present in both eukaryotes and bacteria (25). The activities of DPP4 in saliva and gingival crevicular fluids have been positively correlated with the presence of periodontitis (16, 26). Its activity was also associated with the presence of P. gingivalis in subgingival plaque (26, 27). Similarly, butyric acid, one of the short-chain fatty acids, has been suggested as an indicator for the development and progression of periodontitis (28). Its concentration in gingival crevicular fluid was positively correlated with the severity of periodontitis clinically (17). In oral microbiota, the butyric acid-producing bacteria are mainly *Porphyromonas*, *Fusobacterium*, and *Prevotella*, genera often found in the subgingival plaque (29). Our data showed that the P. gingivalis-enriched biofilms exhibited significantly higher DPP4 activity and produced more butyric acid than Pg0 biofilms, indicating that the P. gingivalis-enriched biofilms resemble the periodontitis-related subgingival plaque functionally. It should be noted that the DPP4 activity and butyric acid production in the control biofilms without P. gingivalis increased with increasing biofilm age. As mentioned above, multiple bacterial species are able to exhibit DPP4 activity and to produce butyric acid (27, 29). Possibly, the growth condition (for example, serum in the growth medium) promoted the growth of DPP4- and butyric acid-producing bacteria and shifted the composition of the microcosm biofilms toward a dysbiotic state. Therefore, these two biomarkers can be used to indicate the dysbiotic state of microbiota *in vitro* but cannot be used to indicate the amount of P. gingivalis in biofilms. Further studies are needed to validate these biomarkers in microcosm biofilms at various ecological states (symbiotic and dysbiotic) and to confirm their applications in distinguishing symbiotic biofilms from dysbiotic biofilms *in vitro*.

In this study, we chose P. gingivalis as the representative periodontal pathogen because it is considered a keystone pathogen to modulate the subgingival microbiome and cause the shift of the microbiome from symbiosis to dysbiosis (30). Interestingly, compared to other oral bacterial species, P. gingivalis is a poor biofilm former when grown alone (31, 32). It requires specific pioneer colonizers (e.g., Streptococcus gordonii and *Veillonella* sp.) or secondary colonizers (e.g., Fusobacterium nucleatum) to attach to a surface or to grow in a biofilm (31, 33). We also grew the P. gingivalis strain ATCC 33277 alone under the same experimental condition (data not shown). The amount of P. gingivalis in the single-species biofilms, quantified by qPCR, was more than 30-fold lower than that in the microcosm biofilms. This further supports that P. gingivalis needs other bacterial species to form biofilms.

It was interesting to observe, in this study, that P. gingivalis was able to overcome the colonization resistance of a preformed biofilm community. The oral microbial community is known for its protective effect against the invasion and colonization of exogenous bacterial species from both oral and nonoral origin in order to maintain community stability (34, 35). Previously, probiotic bacterium Lactobacillus salivarius was found unable to establish itself in a microcosm biofilm when it was added to 24-h or 48-h preformed biofilms. However, it could incorporate into the microbial community when it was added simultaneously with the saliva inoculum (36). The oral bacteria Enterococcus faecalis and Streptococcus mutans were also found to have reduced ability to incorporate in preformed biofilms (37, 38). Our data demonstrated that P. gingivalis was able to integrate into a 24-h preformed biofilm and further proliferated in the biofilm, as the abundance of P. gingivalis (OTU1) increased from 2.7% on day 3 to more than 50% on day 10. Thus, the ability to integrate into an established microbial community is likely a special feature of P. gingivalis, which also strengthens its keystone function in elevating the virulence of the entire microbial community.

As mentioned previously, diverse and pathogen-enriched *in vitro* subgingival microbiota have been established in several studies using subgingival plaque as the inoculum (11, 12, 15). However, the composition of these *in vitro* biofilms varies considerably among studies. For example, the relative abundance of *Porphyromonas* accounted only for 0.5% in 28-day biofilms in the study by Cieplik et al. (15), whereas this number reached 50% in 14-day biofilms in the study by Fernandez et al. (12). Our data were in line with the latter study. The reason for this discrepancy is unclear. Key components in biofilm growth medium, such as hemin, menadione, and serum, were considered to play a critical role in the outgrowth of *Porphyromonas* in *in vitro* biofilms (13, 39). It is known that hemin and menadione are critical for the growth of black-pigmented bacterial species such as P. gingivalis (40). The concentration of serum has been recently suggested to be linked to the enrichment of P. gingivalis in a microbial community. Baraniya et al. (11) observed a high abundance of *Porphyromonas* in the health-associated microbiota and explained that the serum concentration of more than 5% in the growth medium was necessary for this enrichment. However, the growth medium alone cannot explain the discrepancy mentioned above, as all three studies used the same growth medium. It is likely that the amount of *Porphyromonas* in the initial inoculum determines its final relative abundance in a multispecies biofilm. In the study by Baraniya et al. (11) and our study, the initial abundance of *Porphyromonas* was 5.8% or 2.7% (PgL group on day 3), respectively, and its final abundance could reach 30% and 50%, respectively. In the study by Cieplik et al. (15), the abundance of *Porphyromonas* in the inoculum was 1.5%, and only 0.5% of *Porphyromonas* was detected in the 28-day biofilms, even though the growth medium used in this study was optimal for the growth of P. gingivalis. Collectively, the enrichment of P. gingivalis in the multispecies biofilms depends not only on the appropriate growth medium but also on the initial amount of P. gingivalis in the biofilms. In a follow-up study, it would be of interest to explore the minimal amount of P. gingivalis required in an inoculum for the establishment of P. gingivalis-enriched polymicrobial biofilms.

In conclusion, using the easily obtainable saliva from healthy donors as an inoculum, P. gingivalis-enriched biofilm communities were developed in this study with reduced Shannon diversity and altered microbial composition, which corresponds to the pathogen-enriched dysbiotic state in severe periodontitis. Besides P. gingivalis, other bacterial species are also actively involved in the dysbiotic subgingival microbiota, such as Tannerella forsythia and Treponema denticola. In this proof-of-principle study, we started with P. gingivalis. In future research, we will explore the possibility of adding other periodontal pathogens into the saliva-derived microbiota to create various types of periodontitis-related microbiota. Nevertheless, this spiked-microbiome biofilm model circumvents the difficulties encountered when using subgingival plaque as the inoculum and allows the formation of microbiota in the dysbiotic state in a controlled and reproducible manner. This model not only allows for understanding factors that accelerate or hinder the shift from symbiosis to dysbiosis but also provides a useful tool to evaluate strategies that could modulate microbial ecology.

## MATERIALS AND METHODS

### Bacterial strain and growth media.

P. gingivalis strain ATCC 33277 was used in this study. Its stock was stored at −80°C in CryoInstant (VWR, Switzerland) and was streaked onto Trypticase soy agar plates containing 5% sheep blood, 5 μg/ml hemin, and 1 μg/ml menadione (blood agar [BA] plates) for cultivation anaerobically (10% CO_2_, 10% H_2_, and 80% N_2_) to ensure the purity of the colonies based on colony morphology. The colonies on BA plates were used for inoculation (described in detail below). Brain heart infusion broth supplemented with 5 μg/ml hemin and 1 μg/ml menadione (BHIHM) was used for P. gingivalis planktonic culture. All microcosm biofilms were cultured with Thompson medium (TP) (41), which consisted of 7 g/liter Bacto proteose peptone, 3 g/liter Trypticase peptone, 5 g/liter yeast extract, 2.5 g/liter potassium chloride, 0.5 g/liter cysteine hydrochloride, 2.5 g/liter pig gastric mucin, 0.02 M phosphate buffer (pH 8.0), 1 mM urea, 5 mM arginine, 1 mM lysine, 1 mM glycine, 10 μg/ml hemin, 1 μg/ml menadione, and 10% heat-inactivated fetal bovine serum.

### Microcosm biofilm formation.

Pooled saliva from six systemically and orally healthy (i.e., no active oral diseases such as caries and periodontal disease) donors, who had not taken antibiotics within the previous 3 months, was used as the inoculum for the growth of microcosm biofilms. Prior to saliva collection, the donors were asked to refrain from oral hygiene for at least 24 h and abstained from food/drink intake for at least 2 h. The study was approved by the Medical Ethical Committee of the VU University Medical Center Amsterdam (document number 2011/236). The donors were informed about the objectives of the study and have signed an informed consent.

Saliva-derived microcosm biofilms were cultured in the Amsterdam Active Attachment model (AAA model) assembled with hydroxyapatite (HA) discs (9.5 mm diameter; Himed, Old Bethpage, NY, USA) (see “Biofilm Model” in the supplemental material) (42, 43). In detail, the pooled saliva was diluted with fresh TP at a ratio of 1:50 and inoculated into a 24-well culture plate (1.5 ml/well). The plate was then covered with the lid containing HA discs and incubated anaerobically at 37°C. After 24 h, the HA discs with 24-h preformed biofilms were inserted in a new 24-well plate containing 1.5 ml/well of different amounts of P. gingivalis cells (preparation is described below). The biofilm medium was first refreshed after 8 h to remove excess P. gingivalis cells and then refreshed daily.

Colonies of P. gingivalis were collected from BA plates and inoculated into BHIHM broth to grow until the stationary phase (22 to 24 h). The P. gingivalis culture was centrifuged 5,000 × *g* for 10 min at room temperature, and the cell pellets were resuspended in TP medium to various cell densities and added to a new 24-well plate at around 1.5 × 10^7^ CFU/well for PgL, 1.5 × 10^8^ CFU/well for PgM, and 1.5 × 10^9^ CFU/well for PgH. The amount of added P. gingivalis was determined by plating the stationary-phase culture on BA plates after serial dilutions. Based on the colony morphology, the purity of P. gingivalis culture was also confirmed. A control group (Pg0) of TP medium alone without P. gingivalis cells was also included.

On days 3, 7, and 10, microcosm biofilms were harvested and subjected to total viable cell counts and 16S rRNA gene amplicon sequencing. In addition, the amount of P. gingivalis in the microcosm biofilms was quantified by real-time PCR using a P. gingivalis-specific TaqMan probe. Biofilm function, dipeptidyl peptidase IV (DPP4) activity, and butyric acid production were also examined, but only on the samples from day 3 and day 10.

The experiment was repeated 3 times, and there were 4 groups per experiment (Pg0, PgL, PgM, and PgH). In each experiment, each group contained three biofilm samples per time point.

### Total viable cell counts.

HA discs with biofilms were carefully detached from the AAA model with forceps and placed in tubes with 2 ml of cysteine peptone water (CPW; 5 g/liter yeast extract, 1 g/liter of peptone, 8.5 g/liter of sodium chloride, and 0.5 g/liter l-cysteine hydrochloride, pH 7.2). The biofilms were dispersed from the discs by vortexing for 30 s, followed by sonication on ice for 2 min at a 1-s pulse at an amplitude of 40 W (Vibra-Cell; Sonics & Materials Inc., Newtown, CT, USA). The samples were serially diluted and plated on BA plates for total viable cell counts. The remaining sonicated samples were split and stored for DPP4 activity assay and 16S rRNA gene sequencing. The CFU were examined after incubating BA plates anaerobically for 7 days at 37°C.

### DPP4 activity of biofilm cells.

DPP4 activity was measured using glycylprolyl-7-amino-4-methylcoumarin (Gly-Pro-AMC) (AAT Bioquest Inc., CA, USA) as the fluorogenic substrate following the instructions of the manufacturer (44). In brief, biofilm cell pellets were washed once and resuspended with 10 mM Tris buffer (pH 7.6). The resuspensions were mixed with 100 μM Gly-Pro-AMC solution at a 1:1 ratio in a 96-well plate. After 40 min of incubation at 37°C, the fluorescence intensity (FI) of the mixture was measured in a fluorimeter (SpectraMax M2; Molecular Devices, Sunnyvale, CA) with 380-nm excitation and 500-nm emission wavelength. A background control group with substrate but without sample was included to indicate background FI, which was subsequently subtracted from sample FI readings.

### Butyric acid production in the biofilms.

Butyric acid production in the biofilms was determined as the concentration of butyrate in the biofilm spent medium that was collected from the last medium refreshment before biofilm harvesting. The concentration of butyrate was measured with capillary electrophoresis on the Beckman P/ACE MDQ system with UV detection at 230 nm and capillary length of 90 cm in reverse mode (45). Biofilm spent medium was filtered (0.2 μm pore size) and diluted 40× with Milli-Q water for measurement. Sodium salt of butyrate was used to prepare calibration standard solutions. Prior to analysis, 5 μl of 0.1 M KOH and 2 μl of 0.1 M sodium oxalate, which serves as the internal standard, were added per 100 μl of each sample or calibration standard solution. The concentration of butyrate in each sample was calculated from a standard curve generated with known concentrations of butyrate.

### 16S rRNA gene amplicon sequencing and data processing.

To evaluate the microbial composition of biofilms with or without the addition of P. gingivalis, the sonicated biofilm samples were subjected to 16S rRNA gene amplicon sequencing. The samples were centrifuged at 13,000 × *g* for 5 min at 4°C, and the cell pellets were resuspended in DNA-free Tris-EDTA buffer. The genomic DNA (gDNA) of the resuspension was isolated with the Mag minikit (LGC Genomics, Berlin, Germany) following the standard protocol in our lab (15). The sequencing and data processing were performed as described previously (15, 45). Briefly, the V4 hypervariable region of 16S rRNA genes was amplified with barcoded forward and reverse primers. The amplicons were pooled equimolarly, purified, and sequenced using the Illumina MiSeq platform at the VUmc Cancer Center Amsterdam (Amsterdam, The Netherlands) to generate 251-bp paired-end reads. The filtered sequences were clustered into operational taxonomic units (OTUs) at 97% similarity. The representative (most abundant) sequence of each OTU was assigned a taxonomy using the Ribosomal Database Project (RDP) classifier (46) and the SILVA version 132 database (47). To identify the taxonomic names of specific OTUs at the species level, representative sequences of the OTUs were aligned to the expanded Human Oral Microbiome Database version 15.2 by blasting them on the HOMD site (http://www.homd.org) using default parameters. The OTU table was subsampled at an equal depth of 11,500 reads per sample to allow comparisons among the samples.

### qPCR of P. gingivalis.

To determine the amounts of P. gingivalis in the biofilms, a species-specific qPCR was performed, using the isolated gDNA as the sample DNA template, P. gingivalis-specific primers/probe, and LightCycler 480 Probes Master (Roche Diagnostics, Basel, Switzerland) (48). The sequences of the primers and probe are as follows: forward, GCGCTCAACGTTCAGCC; reverse, CACGAATTCCGCCTGC; and probe, Cyan500-CACTGAACTCAAGCCCGGCAGTTTCAA-BBQ. The reaction mixture was analyzed using the LightCycler 480 II (Roche Diagnostics) with the following protocol: preincubation at 95°C for 5 min and then 45 amplification cycles (denaturation at 95°C for 10 s; annealing and extension at 60°C for 20 s). The DNA concentration (nanograms per microliter) of P. gingivalis was calculated based on the standard curve derived from genomic DNA isolated from a pure P. gingivalis culture.

### Data analysis and statistics.

One-way ANOVA followed by the Bonferroni *post hoc* test was performed in SPSS version 25 (SPSS Inc., Chicago, IL, USA) to examine the effects of the addition of P. gingivalis on the total viable cell counts, amount of P. gingivalis DNA, DPP4 activity, and butyric acid production in the biofilms. CFU counts were log_10_ transformed before analysis. Data are presented as mean ± standard deviation. Differences were considered statistically significant if the *P* value was less than 0.01.

For sequencing data analysis, the number of OTUs and the Shannon diversity index were calculated in PAST (PAlaeontological STatistics) version 3.20 (49), and statistical analysis was performed as described above. The OTU table was then log_2_ transformed to normalize the data distribution for principal-component analysis (PCA) in PAST, and statistical differences in the microbial composition were assessed with one-way permutational multivariate analysis of variance (PERMANOVA) based on the Bray-Curtis similarity index. Next, linear discriminant analysis effect size (LEfSe) and network analysis were conducted using a filtered OTU table in which all OTUs with ≤100 reads were removed. Moreover, the data of PgL, PgM, and PgH biofilms at each time point were treated as one group, represented as “Pg group” in the analysis. LEfSe (50) was performed to determine which OTUs were differentially abundant between biofilm with and without the addition of P. gingivalis. Biofilm age was taken as a subclass for LEfSe, and default settings were used, except that the alpha value for all tests was set to 0.01 and pairwise comparisons were performed only among the subclasses with the same name. To assess the microbial copresence or mutual exclusion patterns at the OTU level, a Spearman correlation network was constructed using the CoNet plugin (51) in Cytoscape version 3.8.0 (52). The data matrix was randomized by 100 row-wise permutations. Stringent correlations were defined as an absolute value of Spearman’s rank correlation coefficient *R* greater than 0.65 and significance value *P* of less than 0.01 after adjusting for multiple testing with the Benjamini-Hochberg false discovery rate (FDR) correction.

### Data availability.

The raw sequence and metadata generated has been submitted to the NCBI BioProject database under accession number PRJNA643400.

## Supplementary Material

Supplemental file 1
