# Supplementary material for: Manipulation of Saliva-Derived Microcosm Biofilms To Resemble Dysbiotic Subgingival Microbiota
Source: Appl Environ Microbiol. 2021 Jan 15;87(3):e02371-20. doi: 10.1128/AEM.02371-20 (PMC7848911; doi:10.1128/AEM.02371-20)
Supplement: Supplemental file 1 [file AEM.02371-20-s0001.pdf]

Biofilm model. Saliva-derived microcosm biofilms were cultured in the Amsterdam Active Attachment model (AAA-model) (1, 2). The biofilm model consisted of a custom-made stainless-steel lid with 24 clamps that various substrata can be inserted in. Photos of this model are included in the cited references. For biofilm growth, the lid with assembled substrata was fitted onto a standard 24-well polystyrene plate containing inoculation or growth medium, which was refreshed by transferring the lid onto a new plate with fresh medium. In our study, hydroxyapatite (HA) discs (9.5 mm diameter; HIMED, Old Bethpage, NY, USA) were used as substrata to mimic the structure of tooth surface. The stainless-steel lid together with the assembled HA discs was sterilized by autoclaving before usage.

References

1. Deng DM, Hoogenkamp MA, Exterkate RA, Jiang LM, van der Sluis LW, Ten Cate JM, Crielaard W. 2009. Influence of Streptococcus mutans on Enterococcus faecalis biofilm formation. J Endod 35:1249-52.
2. Exterkate RA, Crielaard W, Ten Cate JM. 2010. Different response to amine fluoride by Streptococcus mutans and polymicrobial biofilms in a novel high-throughput active attachment model. Caries Res 44:372-9.

Table S1. Reproducibility of biofilms from three independent experiments based on Bray-Curtis (BC) similarity [a].

	**BC similarity index (Exp [b]. 1 vs exp. 2 vs exp. 3)**		
	**Day 3**	**Day 7**	**Day 10**
**Pg0**	0.81 (0.03)[c]	0.81 (0.05)[c]	0.75 (0.07)[c]
**PgL**	0.82 (0.03)[d]	0.78 (0.04)	0.80 (0.05)[c]
**PgM**	0.81 (0.03)	0.79 (0.04)	0.79 (0.08)[d]
**PgH**	0.84 (0.03)	0.79 (0.03)	0.80 (0.04)

a. Data were expressed as the mean (standard deviation) of BC pair comparisons: three replicate biofilm samples from each experiment were compared to the three replicate biofilm samples in another experiment respectively.
b. Exp., experiment.
c. One sample was lost in exp. 2 after subsampling of the sequencing data
d. Two samples were lost in exp. 2 after subsampling of the sequencing data
